# Supplementary material for: Ovarian Cancer and Glutamine Metabolism
Source: Int J Mol Sci. 2023 Mar 6;24(5):5041. doi: 10.3390/ijms24055041 (PMC10003179; doi:10.3390/ijms24055041)
Supplement: Supplementary file 1 [file ijms-24-05041-s001.zip › ijms-2215109-supplementary.pdf]

Supplementary Materials

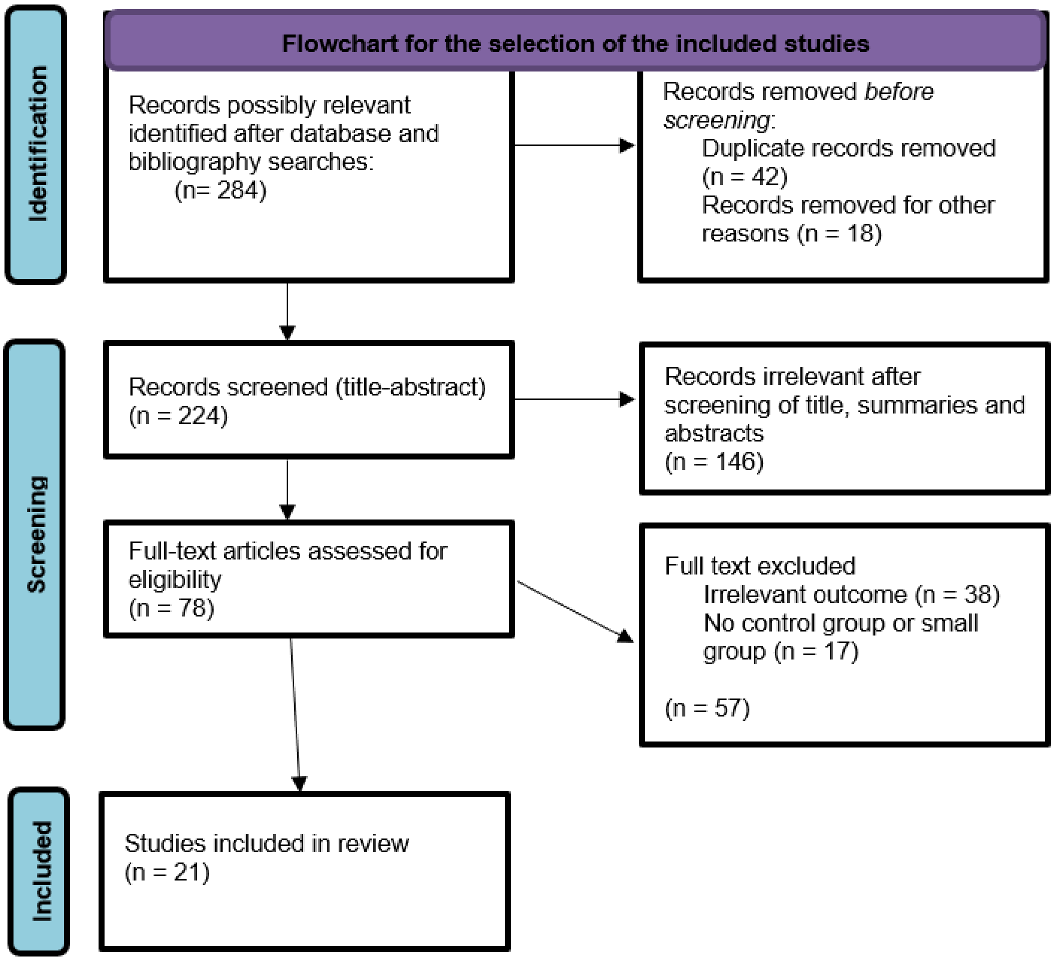

Figure S1. Flowchart of the selection of the included studies.

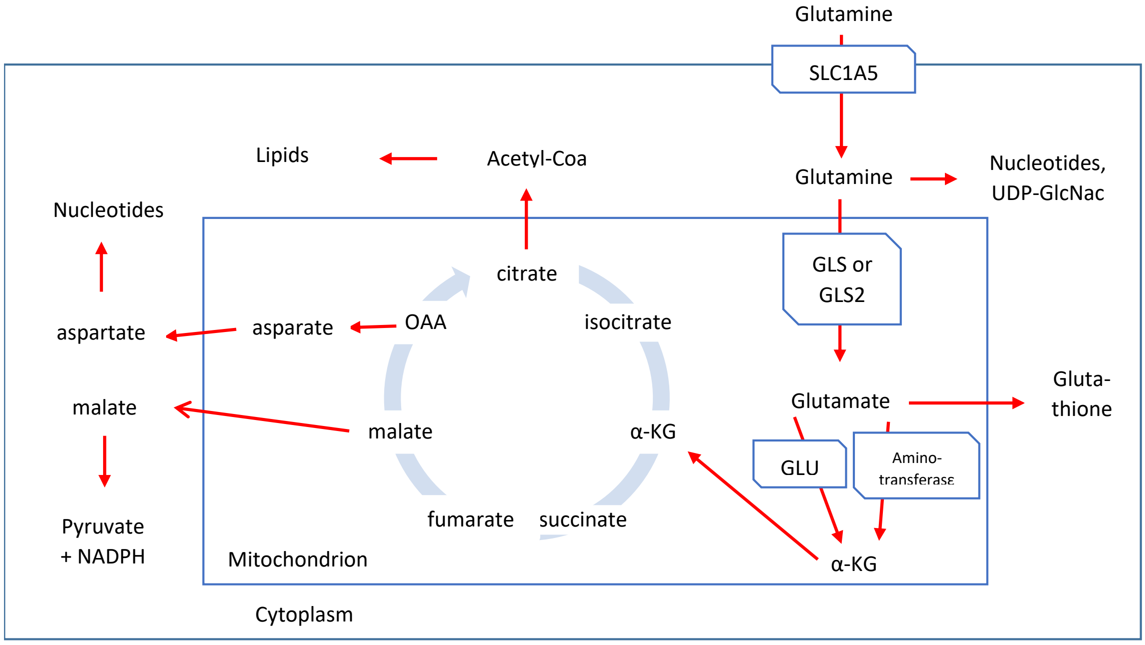

Figure S2. Main metabolic pathways of glutamine. (SLC1A5, solute carrier family 1 member 5; GLS, glutaminase, GLUD, glutamate dehydrogenase; α-KG, α-ketoglutarate; OAA, oxaloacetate; NADPH, reduced nicotinamide adenine dinucleotide phosphate.

Table S1. Overview of the studies on glutamine metabolism and its connection to ovarian cancer.

Reference	Study Design	Target	Study's Target	Result
(Guo et al., 2021)	Case control study	How OC obtains cisplatin (CDDP) resistance.	• CDDP-sensitive ovarian cancer A2780 cells.<br>• CDDP-resistant A2780cis cells.	• ↑ levels of glutamine, glutamate, and GSH in A2780cis cells.<br>• Glutamine deficiency resulted in ↓ glutathione (GSH) concentrations & CDDP resistance in A2780cis cells.
(Yang et al., 2014)	Case control study	Role of glutamine metabolism (via TCA) in cellular growth.	• High- and low-invasive<br>• OVCA cells.	• Link between cancer invasiveness and glutamine concentrations.<br>(High invasive-gln addicted)<br>• Glutamine as a biomarker for patient prognosis.
(Masamha & LaFontaine, 2018)		Effects of combined treatment of the glutaminase inhibitor bis-2-(5-phenyl acetamido-1,3,4-thiadiazol-2-yl) ethyl sulfide (BPTES) with chemotherapy on drug resistant ovarian cancer cells.	Paclitaxel- and cisplatin-resistant cancer cells.	• Monotherapy with BPTES alone resulted in a significant reduction in the ability of glutamine dependent cancer cells to form colonies in a clonogenic assay.<br>• GLS1 isoforms (GAC or KGA) are important for glutamine dependent ovarian cancer survival and should be targeted in metastatic ovarian cancer therapy.
(Tian et al., 2017)	Comparative analysis	Glutamine and glutamate metabolisms in cancer.	Gene expression data of:<br>• cancer tissues vs<br>• normal control tissues	Glutamine generally not involved:<br>• in purine synthesis (except for breast cancer).

			of 11 cancer types.	• in pyridine synthesis (except kidney cancer).<br>• in ATP production.<br>• in asparagine synthesis (except for bladder and lung cancers).<br>• in serine synthesis (except bladder cancer).<br>Glutamine's contribution to nucleotide synthesis is minimal in any cancer.
(Yuan et al., 2015)	Comprehensive analysis	Effects of glutamine on ovarian cancer cell growth<br>(cell cycle progression, apoptosis, cytotoxicity, cellular stress).	The three ovarian cancer cell lines:<br>• HEY<br>• SKOV3<br>• and IGROV-1.	• Each line appears to increase in cell proliferation proportional to the amount of glutamine they receive.<br>• Glutamine increased activity of glutaminase (GLS) and glutamate dehydrogenase (GDH).<br>• Target glutamine metabolism as a therapeutic strategy for ovarian cancer therapy.
(Cluntun et al., 2017)	Review	Glutamine's metabolism in cancer therapy.	Factors influence glutamine's participation in cancer therapy (type of tissue, underlying cancer genetics, micro-environment's tumor, maybe food and host physiology).	Metabolic phenotypes of cancer cells have excessive heterogeneity from those that are highly dependent on catabolism of exogenous glutamine to those that accumulate glutamine via *de novo* synthesis and are self-sufficient for glutamine requirements.
(Yang et al., 2016)	Case control study	Targeting stromal cells might be a potential therapeutic to control communication between the tumor microenvironment (TME) and cancer cells.	Using an orthotopic intra-ovarian mouse model for ovarian carcinoma.	• Metabolic differences between CAFs and normal ovarian fibroblasts (NOFs) include more Gln anabolic pathways in CAFs compared to NOFs.<br>• Dysregulated CAF metabolism induced adaptive mechanisms for harnessing carbon and nitrogen from atypical sources to synthesize Gln in environments where Gln is scarce.<br>• Co-targeting highly expressed stromal glutamine synthetase GS with highly expressed cancer cell glutaminase (GLS) in an orthotopic intra-ovarian mouse model revealed that the metabolic interdependence of CAF and ovarian cancer (OVCA) cells confers a synthetic lethality in tumor and stromal compartments (↓ tumor weight, nodules, and metastasis).
(Kobayashi et al., 2022)	Review	Role of ROS production and antioxidant defense systems in the development of ovarian cancer cells, as possible therapeutic strategies.	Redox homeostasis<br>Antioxidant defense systems (Redox cofactors, antioxidant transcription factors, detoxifying enzymes, and molecular scavengers).	• ROS have a positive and negative impact on cancer evolution, leading to cancer progression (ROS levels below the threshold) or cell death (ROS levels beyond the threshold).

				• Suppress tumors via:<br>↑ ROS generation<br>↑ the antioxidant defense system.
(Leippe et al., 2017)		Metabolite detection assays developed to measure glucose, lactate, glutamate, and glutamine were characterized and evaluated for adaptability to HTS.	Two ovarian cancer cell lines were involved:<br>OVCAR-3 cells, low-invasive and glutamine-independent.<br>SKOV-3 cells, high-invasive and require glutamine for growth.	• Difference in glutamine metabolism between OVCAR-3 and SKOV-3 cells (↑ in OVCAR-3 cells but ↓ in SKOV-3 ones).<br>• OVCAR-3 cells secrete much less glutamate over time than SKOV-3 cells.<br>• Differences in the production of lactate and glutamate by SKOV-3 cells were also identified.
(Hudson et al., 2016)	Meta Analysis	Possible effect of glutamine metabolism on drug resistance to platinum-based chemotherapy for ovarian cancer.	Evaluated two paired cell lines: the cisplatin-sensitive A2780 cell line and its cisplatin-resistant derivative, CP70, together with the cisplatin-sensitive OV 81.2 cell line.	• C-Myc gene expression increases the glutamine dependency of platinum-resistant ovarian cancer cell lines on metabolism.<br>• Glutaminase (GLS) overexpression confers platinum resistance and its inhibition via BPTES re-sensitizing platinum-resistant cells.
(Udumula et al., 2021)	Case control study	Role of immunosuppressive CD11b^{+}Gr1^{+} myeloid cells in ovarian cancer growth, and how the ovarian cancer microenvironment can increase the immunosuppressive ability of these cells.	Intraperitoneal ID8 syngeneic epithelial ovarian cancer mouse model.	• Metabolic fitness of myeloid cells can be increased by increasing glutamine metabolism.<br>• Targeting glutamine metabolism via DLST in immunosuppressive myeloid cells decreases their activity, leading to a reduction in the immunosuppressive tumor microenvironment.
(Wang et al., 2020)	Case control study	Effect of glutamine on the immune function of ovarian cancer mice, treated with paclitaxel.	Fifty (50) SPF female BALB/c mice, divided equally into five groups:<br>• normal control group.<br>• tumor control group.<br>• paclitaxel group.<br>• glutamine group.<br>• combined intervention group.	• Paclitaxel does not improve the immunity of patients during the treatment of ovarian cancer.<br>• Glutamine is an effective immunomodulator.
(Chiu Li et al., 2002)	Review	Deal with toxic effects of the therapeutic substance on normal cells.	Polyanhydride implant (Septacin) containing gentamicin sulfate.	• Achieved high drug concentration at the site of infection while maintaining low systemic drug levels.
(Hu et al., 2010)	Review	p53's role in tumor suppression.	Glutaminase 2 (GLS2) (Mitochondrial glutaminase catalyzing the hydrolysis of glutamine to glutamate).	• p53 increases GLS2 expression.<br>• GLS2 regulates:<br>• Cellular energy metabolism as it induces the production of glutamate and α-ketoglutarate, which, in turn, results in enhanced mitochondrial respiration and ATP generation.<br>• Antioxidant defense function in cells by increasing reduced glutathione (GSH) levels.

				• Decreasing ROS levels, which, in turn, protects cells from oxidative stress.
(Antico Arciuch et al., 2013)		Molecular and metabolic alterations.	Active repression of TCA cycle and OXPHOS in preneoplastic thyrocytes.	• PI3K activation induces, through the inactivation of AMPK, a coordinated repression of the expression of TCA cycle and respiratory genes, which favors aerobic glycolysis at the expense of OXPHOS.
(Kaadige et al., 2009)		Why glucose and glutamine are required for cell growth; MondoA transcription factor.	• Glucose- and glutamine-starved cells.<br>• Glucose-only cells.<br>• Glutamine-only cells.<br>• Glucose plus glutamine cells.	• Consistent with their combined requirement for cell division, neither glucose nor glutamine alone induced pathways important for cell growth or proliferation.<br>• There was no significant overlap in the biological pathways induced by glucose or glutamine alone, indicating that they also have independent roles.<br>• The combination of glucose and glutamine together induced multiple pathways required for cell division.
(E Ó hAinmhire et al., 2014)	Case control study	Expression of wild-type or mutant p53 or the absence of p53 alters ovarian cancer cell response to TGFβ signaling.	Six known ovarian cancer cell lines were analyzed: OVCA 420, OVCA 429, SCOV3, OVCAR 5, OVCA 432, and OVCAR 3.	• Expression of p53 did not alter the ability of the ovarian cancer cells to respond to TGFβ.<br>• p53 status did affect proliferation, migration, and expression of pro-invasive genes.

Table S2. Clinical trials in ovarian cancer targeting glutamine metabolism.

Study Phase	NCT Identifier	Condition Or Disease	Therapy	Study Goal	Current Status
I	NCT03944902	Ovarian cancer-resistant BRCA wild-type ovarian cancer	Glutaminase inhibitor IPN60090 in combination with pembrolizumab	Occurrence of adverse effects	Recruiting
I	NCT05039801	A spectrum of solid tumors, including ovarian cancer	CB-839	Evaluate agent toxicity as demonstrated by the incidence and frequency of treatment-related side effects	Terminated

Table S3. Abbreviations.

Glutamine	Gln	Oxidative phosphorylation	OXPHOS
Glutathione	GSH	Cancer-associated fibroblasts	CAFs
Tumor microenvironment	TME	High-throughput screening	HTS
Glutamate dehydrogenase	GDH	Tricarboxylic acid	TCA circle
